# Growth Curves for Girls with Turner Syndrome

**DOI:** 10.1155/2014/687978

**Published:** 2014-05-15

**Authors:** Fabio Bertapelli, Antonio de Azevedo Barros-Filho, Maria Ângela Reis de Góes Monteiro Antonio, Camila Justino de Oliveira Barbeta, Sofia Helena Valente de Lemos-Marini, Gil Guerra-Junior

**Affiliations:** ^1^Growth and Development Lab, Center for Investigation in Pediatrics (CIPED), School of Medicine, State University of Campinas (UNICAMP), 13083-887 Campinas, SP, Brazil; ^2^Department of Pediatrics, School of Medicine, State University of Campinas (UNICAMP), 13083-887 Campinas, SP, Brazil

## Abstract

The objective of this study was to review the growth curves for Turner syndrome, evaluate the methodological and statistical quality, and suggest potential growth curves for clinical practice guidelines. The search was carried out in the databases Medline and Embase. Of 1006 references identified, 15 were included. Studies constructed curves for weight, height, weight/height, body mass index, head circumference, height velocity, leg length, and sitting height. The sample ranged between 47 and 1,565 (total = 6,273) girls aged 0 to 24 y, born between 1950 and 2006. The number of measures ranged from 580 to 9,011 (total = 28,915). Most studies showed strengths such as sample size, exclusion of the use of growth hormone and androgen, and analysis of confounding variables. However, the growth curves were restricted to height, lack of information about selection bias, limited distributional properties, and smoothing aspects. In conclusion, we observe the need to construct an international growth reference for girls with Turner syndrome, in order to provide support for clinical practice guidelines.

## 1. Introduction

Turner syndrome (TS) is a chromosome abnormality [1], occurring in 1 in 2500 to 1 in 3000 live-born girls [2]. Short stature is one of the most common physical features in girls with TS and growth hormone therapy is recommended for normalization of height [1]. According to Davenport [3], the mean final height in TS girls is 20 cm below the mean of the normal girls. The physiological variations during the growth makes difficult the construction of new height references for TS girls. The World Health Organization (WHO) [4] and the Centers for Disease Control and Prevention (CDC) [5] have constructed growth charts for children and adolescents in the general population, but the accuracy of these curves is questionable for monitoring the growth in TS girls. Saari et al. [6] tested the WHO curves [4] with longitudinal data of TS girls and demonstrated that the growth references of the WHO [4] may have important limitations for monitoring the growth of the children with potential morbidity. Thereby, growth curves for TS girls have been constructed in several countries. However, it is necessary to analyze the methodological and statistical aspects in order to provide accurate information for monitoring of growth in TS girls. Thus, the objectives of this study were to systematically (1) identify existing growth curves for TS girls (2) to evaluate the methodological and statistical quality (3) and to suggest potential growth curves for clinical practice guidelines.

## 2. Methods

### 2.1. Search Strategy

Search strategies were developed together with an expert librarian of the health field. The search was carried out from September to October 2013 in databases: Medline (1950–2013) and Embase (1988–2013). The terms were restricted in English and consulted according to the Medical Subject Headings (MeSH): Turner syndrome, growth chart, growth curve, growth, pattern, standard, reference, value, weight, length, height, velocity, head circumference, body mass index, and BMI, with the possible suffixes. The references of the selected papers were also reviewed. The studies were identified by the electronic search by two independent reviewers. The date of the last search was October 15, 2013.

### 2.2. Study Selection

The search process was developed according to the PRISMA (Preferred Reporting Items for Systematic reviews and Meta-Analyses) method [7]. Two independent reviewers evaluated the potentially relevant studies. Inclusion criteria were (1) original papers, and (2) construction of curves for weight, length/height, growth velocity, head circumference, and BMI. Papers were not excluded by the use of hormonal therapy, sample size, other methodological limitations, and inappropriate statistical analysis.

### 2.3. Data Extraction

The data were extracted according to the international guidelines for the development of growth curves [4]. Discrepancies between reviewers were discussed and resolved by consensus meeting. Data extracted: size sample; number of observations; size sample by karyotype (%); age; type of study; hormone therapy (type, occurrence, and age); spontaneous puberty (percentage, frequency, and age); occurrence of menarche (percentage, frequency, and age); birth year period; growth variable; country; statistical method (mean, standard deviation, percentiles,* z*-scores, smoothing aspects); exclusion criteria; and outcomes.

### 2.4. Data Analysis

Papers that did not provided the total number of observations (*n* ^2^) were subjected to mathematical calculations. Thus, the tables were consulted and *n* ^2^ was calculated (sum of number of observations by age). Furthermore, percentage frequency (%) was calculated to provide the occurrence of puberty, menarche, and karyotypes.

## 3. Results

### 3.1. Study Selection

**Figure 1:**
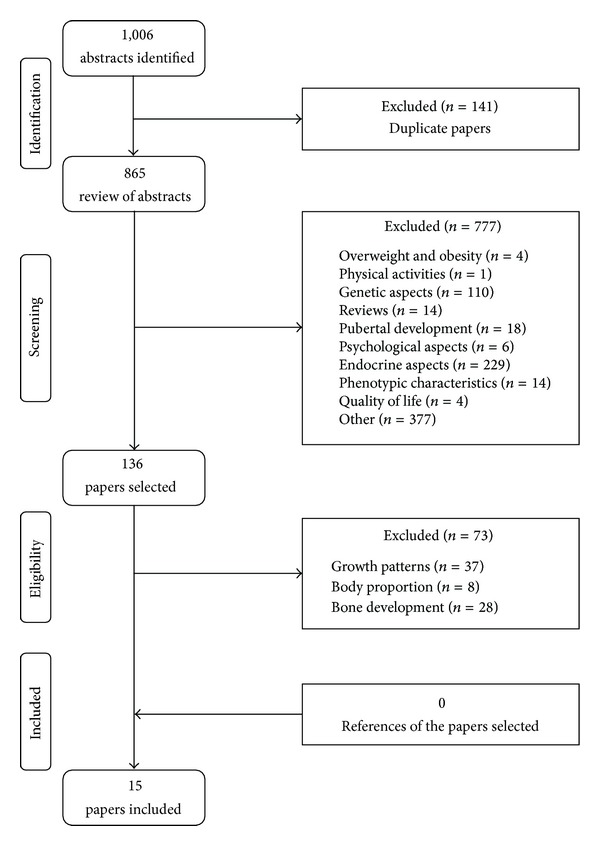
Flowchart of the study selection process.

The search in the electronic databases identified 1,006 studies. The exclusion of papers occurred in three phases: (a) duplicates (*n* = 141); (b) papers that did not evaluate growth charts (*n* = 777); (c) papers on growth pattern, body proportion, and bone development (*n* = 73). The references of the selected papers were not used (*n* = 0). After the eligibility, 15 papers were included in this review [8–22]. The search results are presented in Figure 1.

### 3.2. Characteristics of Studies

#### 3.2.1. Participants

The sample size ranged between 47 and 1,565 TS girls (total = 6,273) aged 0 to 24 y, born between 1950 and 2006. Hormone therapy treatment (estrogen and growth hormone) was observed in girls aged 11.3 to 24.6 y, spontaneous menarche occurrence (aged 11 to 22 y), and spontaneous puberty occurrence (variation: 1.4 to 18.4% of sample)—Table 1. The karyotype 45,X was reported in most studies (Table 2).

#### 3.2.2. Growth Curves

The curves were generated for: weight-for-age; height-for-age; weight-for-height; BMI-for-age; head circumference-for-age; height velocity (cm/year/age) (Table 1). In addition, leg length-for-age and sitting height-for-age [15] also were constructed, but not analyzed in this study. The studies used longitudinal and cross-sectional samples analyzing data collected from 1960 to 2008 (Table 1). The number of anthropometric measurements ranged between 580 and 9,011 (total = 28,915).

#### 3.2.3. Exclusion Criteria

The exclusion criteria applied by the studies were hormone therapy (growth hormone and/or androgen [8, 11, 12, 14, 16, 18–22]; estrogen [12, 18, 19]); diseases (diabetes [10, 11], hypothyroidism [10, 11], hyperthyroidism [11], polyarthritis [10], heart problems [11, 14, 18], and kidney problems [11, 14]); specific karyotypes including the Y chromosome [11, 19]; concomitant autosomal chromosome abnormalities [18]; records that did not include at least two successive annual measurements [15]; spontaneous puberty [16, 18, 20, 21]; gestational age (<37 weeks) [18]; age over 20 years [20]; lack of records about puberty [20]; highly unlikely measurements [20]; and outliers [21].

#### 3.2.4. Types of Curves Generated

The growth curves were constructed using the mean, standard deviation, percentiles,* z*-scores, and smoothing techniques. Seven studies [8–10, 14, 16, 19, 20] provided the number of observations with the respective means and standard deviations by age (Table 3). Suwa [11] showed the means and standard deviations of height at each month in girls aged 0 to 20 y without and with genital bleeding. The age intervals up to the two years were as follows: (a) every one year [8, 9, 11–15, 19, 21, 22]; (b) every 6 months [20]; (c) every 3 months [16–18]. The growth was analyzed according to karyotypes [9–13, 15, 17–21], parents' height [9, 10, 13], hormone therapy [8, 10, 14, 15], spontaneous puberty [9, 12–14, 19], and spontaneous menarche [8, 10, 11, 13, 14]. Percentiles curves were generated for weight-for-age, height-for-age, leg length-for-age, sitting height-for-age, and height velocity [8, 12–15, 18, 20]. The percentiles were generated for the following lines: 3rd, 10th, 25th, 50th, 75th, 90th, and 97th [14, 15, 20]. Other studies (*n* = 7) [9–11, 16, 17, 21, 22] derived the percentiles within the values corresponding to the* z*-score −2 and +2. Román et al. [19] presented only the values of mean and standard deviation by age.

#### 3.2.5. Smoothing Aspects

The smoothing techniques for construction of growth curves were performed in 80% of the studies [8, 11–18, 20–22]. Examples of these methods included (1) 4253H-twice for smoothing the data but without giving specific detail [13], (2) “moving mean” technique to correct the height at each month of age [11], (3) fifth-degree polynomial applied to smooth the mean and standard deviation of height, in order to calculate and select the centile [14], (4) degree polynomial to mean and standard deviation of height, weight-for-age separately for age 0–2 years and for 2–17 years, and weight-for-height [16], (5) splines for each individual height curve to give the individual velocity height [17], (6) LMS methods for BMI curves [16], weight and height curves [20], weight, weight-for-height, and BMI curves [21]. Others studies [8, 12, 15, 18] used smoothing techniques but did not described the exact techniques for constructing the growth curves.

### 3.3. Outcomes

#### 3.3.1. TS Girls versus Normal Girls

The growth references were compared using standard deviation score (SDS) for girls with TS and normal girls, [8, 14, 16–18, 22]. The growth in TS girls was significantly lower in comparison to normal girls (SDS: −0.15 to −2.3).

#### 3.3.2. Hormone Therapy

Most of the studies [8, 11, 12, 14, 16, 18–22] excluded the TS girls that were treated with growth hormone and/or androgens. However, some studies [8–10, 13–15] included the girls that were treated with estrogens, and only three studies [8, 10, 14] analyzed the effects of treatment on the growth of TS girls. There was a significant increase in acceleration of growth during the first year of treatment [8, 14], but did not find significant differences on final height [10].

#### 3.3.3. Karyotypes

The growth was analyzed in different karyotypes. The growth in TS girls with karyotype 45,X was lower in comparison to TS girls with other karyotypes [9, 12]. However, Isojima et al. [21] showed that the stature in children with 45,X was significantly higher than in other karyotypes. Other studies [10, 11, 13, 15, 17–20] found no significant differences.

#### 3.3.4. Parents' Height

Some studies [9, 10, 13] analyzed the relation between parental height and the height of girls with TS. Massa et al. [9] found significant positive correlation between the parental height and the height of girls with TS (*r* = 0.39). In the study of Naeraa and Nielsen [10], there was a significant correlation between mid-parental height and the adult height of girls with TS (*r* = 0.67; *n* = 65). Bernasconi et al. [13] demonstrated tendency towards higher girls size which can be attributable to the higher height means of mothers and fathers (TS: 146.3 ± 3.4 cm, mid-parental: 169.5 ± 3.4 cm versus TS: 139.9 ± 6.5 cm, mid-parental: 159.4 ± 3.7 cm).

#### 3.3.5. Spontaneous Puberty and Menarche

Some studies analyzed the effects of spontaneous puberty [9, 13, 14, 19] and menarche occurrence [8, 11, 13, 14] on the final height in girls with TS, but found no significant differences with the induced puberty by hormone replacement, and the absence of menarche. In the study of Massa et al. [9], the TS girls aged 11 to 13 y with spontaneous puberty presented higher height and height velocity in comparison to TS girls with induced puberty.

#### 3.3.6. Study Limitation

With regard to the reported limitations, only two studies [20, 21] described the methodological limitations for the construction of growth curves in TS girls. They reported the limitations with regard to selection bias, retrospective data, sample size in specific ages, cross-sectional design, and occurrence of puberty.

## 4. Discussion

Growth curves are an important tool to evaluate child health [4, 27, 28]. In 2006, the WHO constructed new curves to follow child growth among diverse ethnic groups, socioeconomic conditions, and feeding mode [29]. However, the growth patterns of children with various diseases were not considered in international references.

Growth curves for TS girls were constructed in different countries. In this review, we describe the main methodological and statistical implications in each study. The curves have important strengths and weaknesses for application in the clinical practice. Some studies analyzed the effects of some confounder variable on the growth in girls with TS such as hormone therapy, spontaneous puberty, karyotypes, and mid-parental height. However, the relationship among the confounder variable and the acceleration or delays of growth appears to vary little in these studies. Length/height curves were found in all studies as well as height velocity in some studies. However, most did not generate curves for weight-for-age, head circumference-for-age, weight/height-for-age, and BMI-for-age. Although the TS girls have different genetic and phenotypic variations, the curves for weight, head circumference, and BMI should be more explored such as the curves of El-Bassyouni et al. [22], Isojima et al. [21], and Rongen-Westerlaken et al. [16].

The stature has increased in the last decades in general population. Isojima et al. [21] demonstrated that the adult height in TS girls followed in the studies of Suwa et al. [11], Isojima et al. [20], and Isojima et al. [21] was 138.2 cm, 141.2 cm, and 141.3 cm, respectively. Most studies followed the TS girls from 1950 to 1990, but did not analyze the growth in different time periods, and more than 50% of studies did not report the birth year period. Thus, we recommend the analysis of secular trend for construction of new growth curves for TS girls.

A strength observed in most studies was the number of observations for construction of curves. Considering the incidence of 1 : 2,500–3,000 girls born alive, the sample size appears to be representative, although the sample calculation is not indicated in the studies. The age interval was not thoroughly analyzed, and few studies used age interval every 3 months. It is difficult to precise the fitting of the curves from birth to 2 years of age. Another strength of the studies is that the girls treated with growth hormones or androgens were excluded of the samples. However, due to the use of retrospective data, there may be several cases omitted because of lack of information in the medical records. In addition, the selection bias may suggest a data bias in some situations, according to limitation reported by Isojima et al. [20]. With regard to environmental factors, no studies evaluated breastfeeding, lactation support, feeding mode, socioeconomic condition, maternal health, pediatric care, among others. de Onis et al. [30], demonstrated that the control of the environmental factors was necessary for construction of new curves as well as the selection of appropriate statistical methods for sample selection, data collection, and analysis.

A bias that is difficult to analyze is the karyotype, because often the information is not given. Over the decades, the techniques of realization of karyotype have improved and the results were significantly modified, decreasing the percentage of 45,X cases and increasing mosaicisms and structural aberrations of the sexual chromosomes [31].

For the construction of the curves, several statistical methods were used such as mean, standard deviation, percentile,* z*-score, and smoothing techniques. The first strength was the number of observations with means and standard deviations provided by most studies, thus allowing a greater accuracy when carrying out the comparisons between the various studies. The main statistical limitation observed in studies was the absence of important statistical methods for the construction of curves such as distribution and smoothing aspects. On the other hand, most studies used percentiles or* z*-score corresponding to −2 and +2. The distribution aspects of the anthropometric data should be analyzed as well as the estimation of percentiles related to age [32]. With regard to smoothing of curves, few studies have applied the LMS method to evaluate asymmetry, median, and data variability of Cole and Green [33]. The lack of smoothing techniques can lead to the construction of irregular curves, regardless of the use of large samples [32]. Moreover, Cole [34] shows that mean and SD for normal data are commonly used for the construction of curves as well as the LMS method for the treatment of asymmetry. According to the WHO [4], 30 statistical methods were reviewed for the construction of the 2006 growth curves. This involved the Box-Cox-power-exponential (BCPE), with curve smoothing by cubic splines. This demonstrates that the appropriate choice of statistical methods should be performed to construct growth curves. Thus, we suggested the examination of existing methods that will allow identifying the best statistical technique for construction of the growth curves in TS girls.

The strengths and limitations of growth curves should be carefully analyzed for clinical application in girls with TS. The main limitation for global use is their origination in a specific country. However, for countries that lack their own growth curve, we believe that the curves of Rongen-Westerlaken et al. [16] appear to be more suitable for clinical applications in children up to 2 years old mainly because the authors used age intervals every 3 months, generated curves for height, weight, BMI, and height velocity, and used high sample size. Moreover, for children above 2 years, we recommend the curves of Isojima et al. [21] for growth monitoring because the authors used high sample sizes and age intervals every 6 month up 20 years old. In addition, they considered secular trend, selection bias, and appropriate statistics.

## 5. Conclusion

In the studies on growth curves in girls with TS, we observe the need to construct new curves for different ethnics group. For this, an important step in the construction of the new curves would be to analyze carefully the methodological and statistical aspects. Furthermore, for global use, we recommend a joint effort to produce a growth curve in TS girls for universal applicability. Finally, the findings of this review provide support for clinical practice guidelines for the monitoring of growth in girls with TS.

## Figures and Tables

**Table 1:** Characteristics of the studies on growth curves.

Study	Age (years)	Type (study)	HT (years)	SP %^e^ (years)	SM %^e^ (years)	Birth^f^	L/H	W	HC	W/H	BMI	HV	Country
Lyon et al. 1985 [8]	1–20+	Mixed	Included >15	h	8 (13–17)	h	Yes	No	No	No	No	No	g
Massa et al. 1990 [9]	0–22	Mixed	Included 11.3–18	17 (11.1–15)	10 (13.7–15.2)	h	Yes	No	No	No	No	Yes	Belgium
Naeraa and Nielsen 1990 [10]	7–20^a^	h	Included 13.2–24.6	h	9 (13–15)	1955–66	Yes	No	No	No	No	Yes	Denmark
Suwa 1992 [11]	0–20	Mixed	Excluded	h	3.3 (11–14)	1955–89	Yes	No	No	No	No	Yes	Japan
Haeusler et al. 1992 [12]	0–16	Mixed	Excluded	18.4 (no/age)	h	1961–81	Yes	No	No	No	No	Yes	Austria
Bernasconi et al. 1994 [13]	0–21	Mixed	Included (>13)	1.4 (20.8)	4.4 (20.2)	1950–90	Yes	No	No	No	No	No	Italy
Garcia Rudaz et al. 1995 [14]	0–22	Mixed	Included 14 ± 1.2	12.7 (9–14)	h (11–16.1)	h	Yes	No	No	No	No	Yes	Argentina
Sempe et al. 1996 [15]	0–20^b^	h	Included	h	h	h	Yes	No	No	No	No	Yes	France
Rongen-Westerlaken et al. 1997 [16]	0–20	h	Included 12.8–20.1^d^	Excluded	h	h	Yes	Yes	No	Yes	Yes	Yes	Netherlands Denmark Sweden
Even et al. 2000 [17]	0–3	Long	h	h	h	h	Yes	No	No	No	No	Yes	Israel
Davenport et al. 2002 [18]	0–8	Long	Excluded	Excluded	h	h	Yes	No	No	No	No	Yes	USA
Román et al. 2002 [19]	0–18+	h	Excluded	10 (no/age)	h	>1968	Yes	No	No	No	No	No	Chile
Isojima et al. 2009 [20]	0–20	Cross	Excluded	Excluded	h	1970–02	Yes	No	No	No	No	No	Japan
Isojima et al. 2010 [21]	0–20	Cross	Excluded	Excluded	h	1970–06	Yes	Yes	No	Yes	Yes	Yes	Japan
El-Bassyouni et al. 2012 [22]	6–24^c^	h	Excluded	h	h	h	Yes	Yes	Yes	No	Yes	No	Egypt

HT: hormone therapy; SP: spontaneous puberty; SM: spontaneous menarche; L/H: length/height; W: weight; HC: head circumference; W/H: weight/height; BMI: body mass index; HV: height velocity; ^a^Height curve (7.5–17.5 years). ^b^Reference curves (1–21 years); ^c^6 months to 24 years; ^d^Age (10 and 90 percentile); ^e^Percentage frequency; ^f^Birth year period; ^g^Data derived from studies (Germany—Pelz et al. [23]; Germany—Ranke et al. [24]; Finland—Lenko et al. [25]; France—Rosenberg and Tell [26]); ^h^Data not available.

**Table 2:** Size sample and karyotypes of the studies.

Study	*n* ^a^	*n* ^b^	Karyotype (%)				
			45,X	45,X/46, XX	46,X, i(Xq)	45,X/46,X, i(Xq)	Others
Lyon et al. 1985 [8]	366	1517	61.8^e^	16.9^e^	4.1^e^	4.6^e^	12.6^e^
Massa et al. 1990 [9]	100	637	47	7	5	13	28
Naeraa and Nielsen 1990 [10]	78	1174	63	3	c	8	26
Suwa 1992 [11]	704	6255	36.1	c	9.8	0	54.1
Haeusler et al. 1992 [12]	141	580	67.3	7	2.8	9.2	13.7
Bernasconi et al. 1994 [13]	772	c	56.3	9.8	7.3	10.9	15.7
Garcia Rudaz et al. 1995 [14]	254	2308	47	15	7	11	20
Sempe et al. 1996 [15]	167	708	58.1	31.7	c	c	10.2
Rongen-Westerlaken et al. 1997 [16]	598	9011	c	c	c	c	c
Even et al. 2000 [17]	47	c	48.9	c	23.4	c	27.7
Davenport et al. 2002 [18]	112	1146	57.1	12.5	4.5	c	25.9
Román et al. 2002 [19]	83	668	60	25	2	9	4
Isojima et al. 2009 [20]	1447	1447^d^	29.9	6	8.8	21.4	33.9
Isojima et al. 2010 [21]	1565	5772	29.6	6.1	8.8	21	34.5
El-Bassyouni et al. 2012 [22]	93	c	59.1	40.9	c	c	c

Total	6273	28915					

*n* ^a^: number of subjects; *n* ^b^: number of observations.

^c^Data not available.

^d^Mentioned in Isojima et al. 2010 [21].

^e^Data derived from studies (Pelz et al. [23]; Ranke et al. [24]; Lenko et al. [25]; Rosenberg and Tell [26]).

**Table 3:** Number of observations, means, and standard deviations (SD) for height in girls with Turner syndrome.

Age (y)	Lyon et al. 1985 [8]			Massa et al. 1990 [9]			Naeraa and Nielsen 1990 [10]			Garcia Rudaz et al. 1995 [14]			Rongen-Westerlaken et al. 1997 [16]			Román et al. 2002 [19]			Isojima et al. 2010 [21]		
	*n*	Mean	SD	*n*	Mean	SD	*n*	Mean	SD	*n*	Mean	SD	*n*	Mean	SD	*n*	Mean	SD	*n*	Mean	SD
0	—	—	—	23	48	1.9	—	—	—	18	46.4	3.5	213	47.9	2.8	42	46.8	2.1	338	—	—
1	81	63.8	6.8	8	69.3	3.3	—	—	—	19	65.3	3.9	182	70.0	2.5	44	73.0	3.9	240	68.79	2.83
2	52	77.5	3.5	11	79.4	3.4	—	—	—	18	77.1	4.1	223	80.8	3.0	32	80.0	4.2	208	77.62	3.03
3	64	84.2	4.7	13	87.2	4.4	—	—	—	18	85.7	4.1	234	87.1	3.5	50	85.5	4.7	364	84.25	3.17
4	58	91.1	5.5	17	91.1	3.6	—	—	—	23	92.2	4.2	247	94.0	3.6	34	91.4	4.2	376	89.56	3.29
5	66	96.5	5.0	19	97.8	3.8	—	—	—	33	97.4	4.2	226	99.6	4.4	61	96.7	4.0	393	94.66	3.42
6	79	103.1	6.4	27	102.4	3.8	—	—	—	42	101.8	4.4	249	104.7	4.2	25	102.1	5.6	447	99.86	3.59
7	77	106.0	5.0	27	106.8	4.1	32	108.9	5.4	52	105.9	4.6	260	109.4	4.5	52	106.2	7.6	469	104.96	3.80
8	83	111.4	6.6	28	110.5	4.3	63	113.6	4.6	55	109.8	4.9	259	114.0	4.7	27	108.9	5.8	477	109.63	4.02
9	97	115.0	5.7	29	115.1	4.4	66	117.8	4.7	67	113.8	5.2	262	118.0	4.9	64	112.3	4.2	557	114.27	4.25
10	93	119.5	6.4	38	120.1	4.5	69	121.6	4.8	82	117.8	5.5	263	121.8	4.8	31	116.7	5.1	511	118.82	4.48
11	84	122.8	6.2	33	124.8	5.2	69	125.4	4.9	86	121.7	5.8	252	125.8	4.9	89	120.2	5.5	413	123.14	4.70
12	105	126.5	5.9	41	128.4	5.4	68	129.0	5.1	91	125.5	6.0	233	130.0	5.3	68	122.8	5.7	308	127.25	4.89
13	85	130.7	5.8	44	132.5	6.1	69	132.4	5.0	90	129.0	6.2	224	133.4	5.6	71	125.8	7.2	209	131.12	5.07
14	85	132.6	5.9	48	135.9	5.9	66	135.8	5.1	93	132.1	6.2	178	136.5	5.8	32	130.3	7.9	163	134.13	5.21
15	84	135.8	5.7	49	138.2	6.2	60	138.6	5.0	93	134.5	6.0	135	139.9	5.8	30	133.5	9.0	117	136.13	5.31
16	84	138.6	5.4	52	140.0	5.7	49	140.2	5.1	87	136.3	5.7	107	140.8	6.0	—	—	—	82	137.64	5.38
17	52	140.6	6.0	43	141.6	6.1	34	142.3	4.8	77	137.4	5.4	72	141.8	5.1	—	—	—	42	138.76	5.43
18	26	143.4	6.3	33	142.5	6.0	—	—	—	62	137.8	5.1	45	143.7	5.2	—	—	—	21	139.51	5.46
